# Chronic spontaneous epidural hematoma in the lumbar spine with cauda equina syndrome and severe vertebral scalloping mimicking a spinal tumor: a case report

**DOI:** 10.1186/s12891-022-05463-x

**Published:** 2022-05-30

**Authors:** Shusaku Fukatsu, Satoshi Ogihara, Hiroki Imada, Satoshi Ikemune, Jun-Ichi Tamaru, Kazuo Saita

**Affiliations:** 1 Department of Orthopaedic Surgery, Saitama Medical Center, Saitama Medical University, 1981 Kamoda, Kawagoe, Saitama, 350-8550 Japan; 2 Department of Pathology, Saitama Medical Center, Saitama Medical University, 1981 Kamoda, Kawagoe, Saitama, 350-8550 Japan

**Keywords:** Cauda equina syndrome, Chronic epidural hematoma, Spinal tumor, Lumbar spine, Vertebral scalloping, Magnetic resonance imaging, Diagnosis

## Abstract

**Background:**

Chronic spinal epidural hematomas (SEHs) are rare clinical entities. SEH with vertebral scalloping is extremely rare, with only a few cases having been reported to date. We report a unique case of spontaneous chronic SEH in the lumbar spine with severe vertebral scalloping mimicking an epidural tumor.

**Case presentation:**

A 71-year-old man presented with a 2-month history of lumbar pain and a 3-week history of paresthesia and pain in the right lower extremity, hypesthesia in the perineal and perianal regions, and bladder dysfunction. Computed tomography following myelography revealed an epidural mass lesion on the right side that compressed the dural sac and was associated with severe bony scalloping on the posterior wall of the L4 vertebral body. Magnetic resonance imaging (MRI) on T1- and T2-weighted images revealed a space-occupying lesion with heterogeneous intensity, and T1-gadolinium images showed an intralesional heterogeneous enhancement effect. A tumoral lesion in the spinal canal was suspected, based on preoperative imaging; therefore, a total spinal tumor resection was planned. Intraoperative findings revealed that the brownish lesion adhered to the dura and epidural tissues in the spinal canal, and the space-occupying mass in the scalloped cavity of the posterior wall of the L4 vertebra was encapsulated in red-brownish soft tissues. The lesion was totally resected in a piecemeal fashion, and pathological examination revealed a mixture of tissues that contained a relatively new hematoma with hemoglobin, as well as an obsolete hematoma with hemosiderin and amyloid deposits. The mass was diagnosed as a chronic epidural hematoma with recurrent hemorrhage. The postoperative course was uneventful, and the preoperative neurological symptoms immediately improved.

**Conclusions:**

The preoperative diagnosis of chronic SEHs is challenging, as MRI results may not be conclusive, particularly in patients with scalloping of bony structures. Thus, chronic SEHs should be considered as a differential diagnosis in cases of suspected tumoral lesions in the spinal canal. To the best of our knowledge, this is the first reported case of acute exacerbation of chronic SEH with cauda equina syndrome and severe vertebral scalloping.

## Background

Spinal epidural hematomas (SEHs) are uncommon clinical entities, accounting for only 0.3–0.9% of all space-occupying lesions in the spinal canal [1, 2]. Chronic SEHs are most frequently located in the lumbar spine [1, 3, 4] and are very rare, accounting for 0.1% of all SEHs [5]. A preoperative imaging diagnosis of chronic SEHs is often not possible, as the results of computed tomography and magnetic resonance imaging (MRI) may be inconclusive [1, 4]. Chronic SEHs with vertebral scalloping are extremely rare. To the best of our knowledge, only two cases have previously been reported [2, 5]. Herein, we report a unique case of spontaneous chronic SEH in the lumbar spine with severe vertebral scalloping and acute cauda equina syndrome, which was initially misdiagnosed as an intraspinal tumor.

## Case presentation

A 71-year-old man presented to our hospital with a 2-month history of lumbar pain and a 3-week history of paresthesia with pain in the right lower extremity, hypesthesia in the perineal and perianal regions, bladder dysfunction (frequent urination and a feeling of residual urine), and intermittent claudication. He had a history of cerebral infarction 8 years prior and was treated with antiplatelet therapy (clopidogrel). All blood and coagulation tests (platelet count, prothrombin time, and partial thromboplastin time) revealed normal values. There was no history of major or minor spinal trauma. On physical examination, the patella tendon reflex was normal bilaterally. The right Achilles tendon reflex was diminished, and the left Achilles tendon reflex was normal. Babinski’s sign was absent bilaterally. He had no motor weakness in his bilateral extremities.

**Fig. 1:**
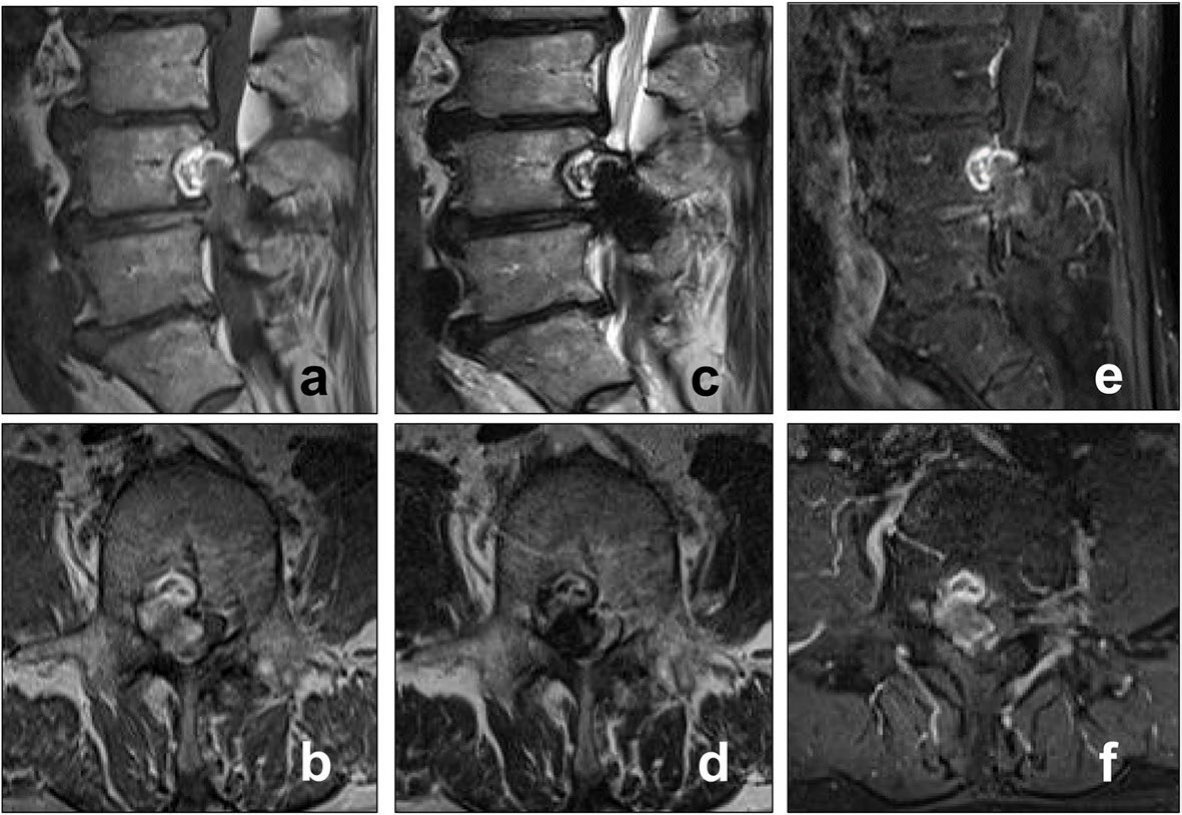
Magnetic resonance imaging (MRI) findings. **a** T_1_-weighted MRI sagittal and (**b**) axial images at the L4 vertebral level, showing the heterogeneous intensity of the space-occupying lesion. **c** T2-weighted MRI sagittal and (**d**) axial images at the L4 vertebral level, showing the heterogeneous intensity of the space-occupying lesion. **e** T1-weighted gadolinium-enhanced MRI sagittal and (**f**) axial images at the L4 level, revealing the intralesional heterogeneous enhancement effect of the space-occupying lesion

MRI performed before hospitalization revealed a space-occupying lesion that extended from the right intravertebral region to the intraspinal canal at the level of the L4 vertebra on the right side. The mass lesion in the spinal canal had heterogeneous intensity on T1-weighted images and low intensity on T2-weighted images. Gadolinium-enhanced T1-weighted images showed a heterogeneous enhancement effect in the lesion (Fig. 1a–f). Based on the preoperative imaging findings of posterior vertebral scalloping and heterogeneous signal intensity on MRI, the space-occupying lesion in the spinal canal was initially suspected to be a benign epidural or cauda equina tumor (e.g., schwannoma).

**Fig. 2:**
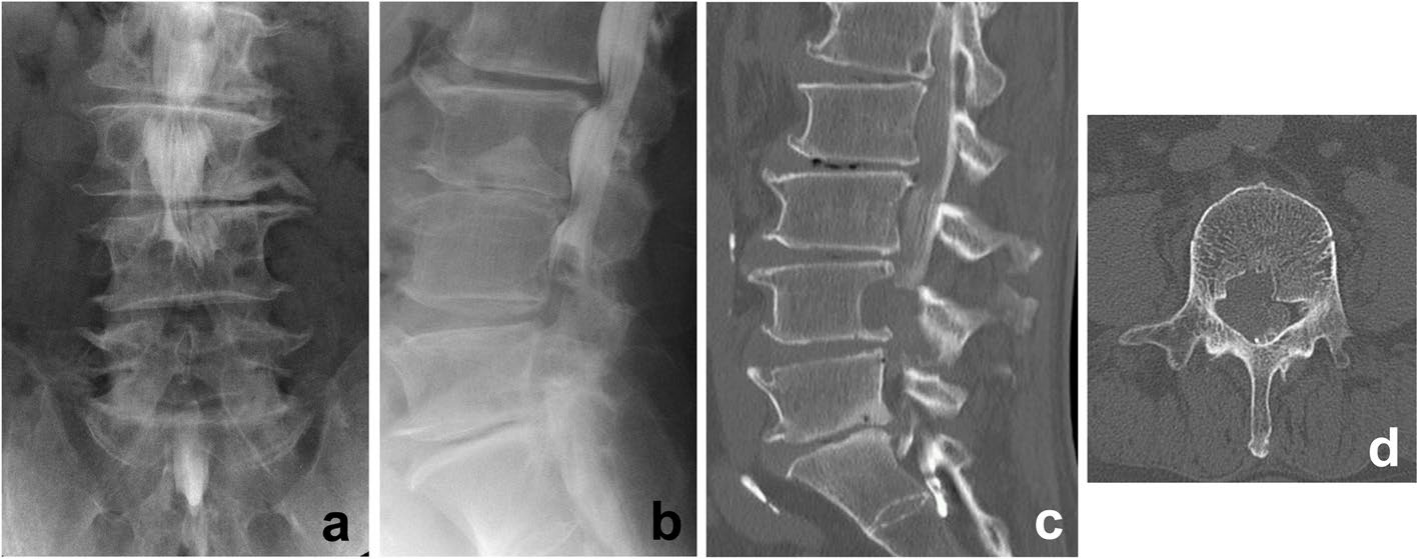
X-ray and computed tomography (CT) findings. **a** Anteroposterior and (**b**) lateral preoperative X-ray images (obtained following myelography) of the lumber spine, revealing the right-side dominant contrast defect of the spinal subarachnoid space at the L3–4 level. **c** CT images of the right parasagittal and (**d**) axial aspects at the level of the L4 vertebra (following myelography), revealing severe scalloping at the right side of the L4 vertebral posterior wall

After admission, myelography revealed a contrast defect of the spinal subarachnoid space at the L4–L5 level predominantly on the right side (Fig. 2a, b). Computed tomography following myelography revealed a space-occupying lesion in the spinal canal on the right side, with bone scalloping over the posterior wall of the L4 vertebral body. Sclerotic bone was observed on the surface of the scalloped region (Fig. 2c, d).

**Fig. 3:**
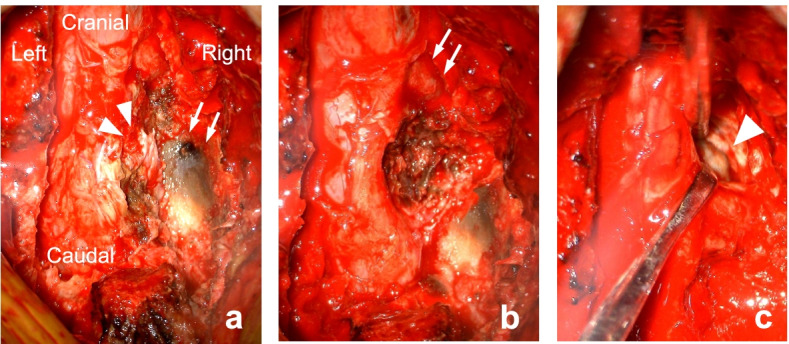
Intraoperative photographs. **a** An encapsulated mass lesion (suspected old hematoma) is observed in the epidural space beneath the ligamentum flavum at the right L4–5 level (arrowheads) after the L4–5 laminectomy and the right L4 inferior facetectomy. Arrows indicate the cartilage surface of the right L5 superior facet that was blackened, presumably by hemosiderin deposits. **b** Photograph taken following the removal of the dark-brownish mass lesion (suspected old hematoma). Arrows indicate the right L4 nerve root. **c** The encapsulated reddish-brownish mass (arrowhead) is observed in the cavity of the scalloped area at the posterior wall of the L4 vertebra, with the right L4 nerve root pulled to the caudal side with a spatula

**Fig. 4:**
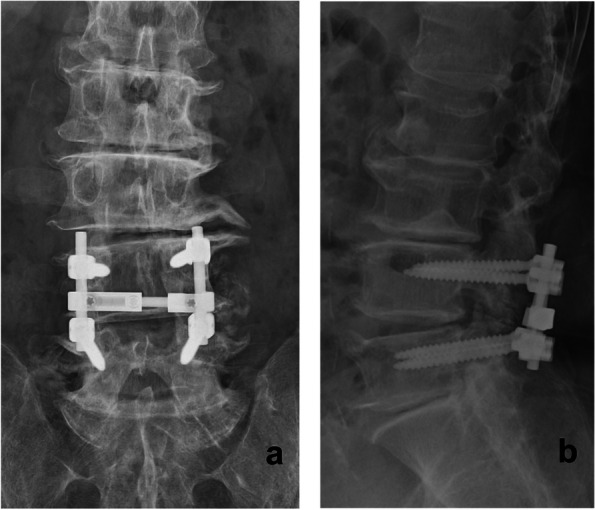
Postoperative (**a**) anteroposterior and (**b**) lateral radiographs of the lumbar spine. Pedicle screws are shown inserted in L4 and L5 bilaterally

We performed a posterior lumbar surgery after stopping clopidogrel administration for 2 weeks for normalization of blood coagulation. A L4–5 laminectomy and right L4–5 facetectomy were performed to allow wide exploration and facilitate a total spinal tumor resection. Intraoperative findings revealed that the surface of the right L4–5 facet cartilage was blackened; prior intra-articular bleeding was suspected (Fig. 3a). The intraspinal brown mass adhered to the epidural tissues, resembling an old hematoma (Fig. 3b). In addition, the red-brownish mass in the scalloped space of the right posterior wall of the L4 vertebra was soft and encapsulated (Fig. 3c). The epidural mass lesion was resected in a piecemeal fashion. An L4–L5 instrumented fusion with local bone grafting was subsequently performed at the left L4–L5 facet (Fig. 4a, b).

**Fig. 5:**
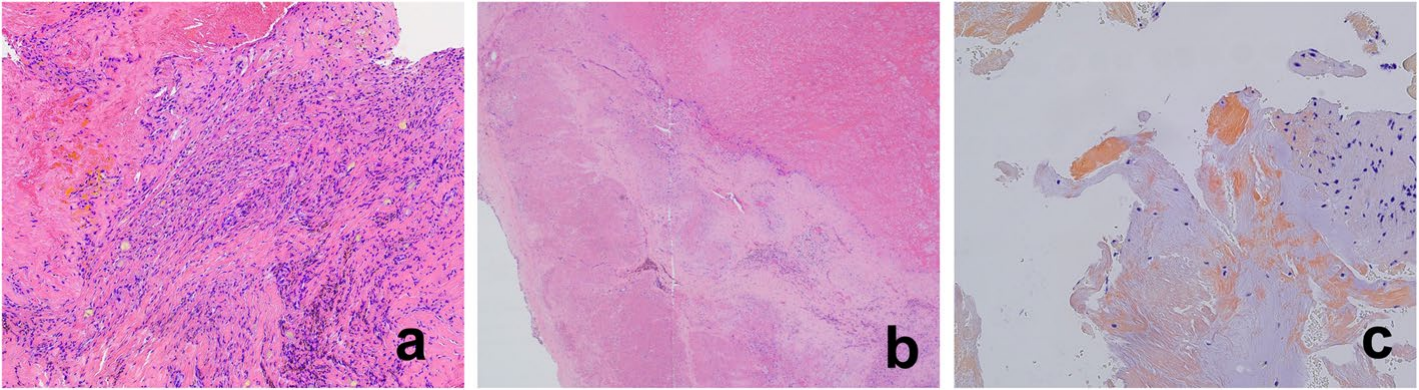
Histological findings. **a** Hemorrhagic change indicated by granulation tissue (with hemosiderin deposition) and focal red blood cells (with fibrin) within the intravertebral scalloped lesion (hematoxylin and eosin staining). **b** Degeneration of the ligamentum flavum within the epidural tumorous lesion and the formation of granulation tissue in an adjacent region (hematoxylin and eosin staining). **c** Direct fast Scarlet stain showing the focal deposition of amyloid within the scalloped and epidural lesion. Green birefringence was confirmed with a polarized filter. These findings indicate a chronic epidural hematoma, which is a non-neoplastic change

Histological examination of the intravertebral scalloped lesion revealed granulation tissue with a blood clot and hemosiderin deposition, as well as an adjacent focally degenerated ligamentum flavum (Fig. 5a). The ligamentum flavum within the epidural tumorous lesion was degenerated with granulation tissue (Fig. 5b). The amyloid was focally deposited within the scalloped and epidural lesion (Fig. 5c). Vascular proliferation suggestive of hemangioma or arteriovenous malformation was not observed. The excised lesion was diagnosed as a chronic epidural hematoma without neoplastic changes.

**Fig. 6:**
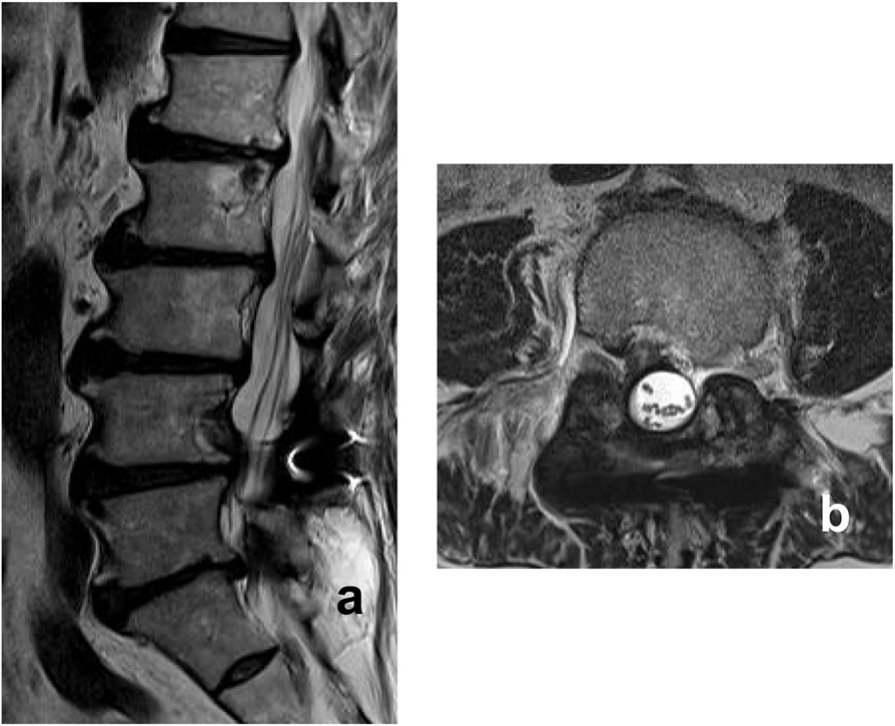
Postoperative magnetic resonance imaging (MRI) findings. **a** T2-weighted MRI of the sagittal and (**b**) axial aspects at the L4 vertebral level show the disappearance of the epidural space-occupying lesion at 2 years postoperatively

The patient’s preoperative neurological symptoms (paresthesia with pain in the right lower extremity, hypesthesia in the perineal and perianal regions, bladder dysfunction, and intermittent claudication) improved immediately after the operation. He was discharged on the 11th postoperative day and was able to walk without aid. The patient was able to resume his previous job delivering newspapers at 2 months postoperatively. Good clinical outcomes were maintained at the final 2-year postoperative follow-up, with MRI confirming the disappearance of the epidural space-occupying lesion (Fig. 6a, b).

## Discussion and conclusions

SEH can be classified as idiopathic, spontaneous, or secondary, based on its pathogenesis [1, 2]. Idiopathic SEHs are not attributed to any specific risk factors. While spontaneous SEHs do not have a clear etiology, they are associated with factors such as anticoagulation therapy, minor trauma, hypertension, and Paget’s disease [1, 2]. In contrast, secondary SEHs have specific etiologies, including coagulopathies, vascular malformations, neoplasms, major trauma (with or without fracture), and medical procedures [1, 2]. The majority of chronic SEHs are either idiopathic or spontaneous. In the present case, we made a diagnosis of spontaneous SEH associated with anticoagulant use (clopidogrel).

The pathophysiology of chronic idiopathic or spontaneous SEH is not clear. One hypothesis is the rupture of Batson’s plexus due to variations in abdominal or thoracic pressure [5–7]. Batson’s plexus is a confluence of valveless veins that run longitudinally; they lie largely on the dorsal surface of the peridural membrane and penetrate it at several points to enter the vertebral body [5]. Chronic SEH may also be caused by the bleeding of small vessels within the degenerated ligamentum flavum that is associated with mechanical stress on the ligamentum flavum and/or a rise in blood pressure. This may explain why this type of hematoma is exclusively located on the dorsal or dorso-lateral aspect [1, 8]. In addition, Graziani et al. [9] reported that some spontaneous hematomas could be due to an epidural cavernous hemangioma. Nevertheless, in the present study, the pathological examination of resected specimens was unable to identify tissues derived from the hemangioma.

Previous studies have reported that chronic SEHs often result in a lumbar radiculopathy with a chronic clinical course [1, 8]. To date, only a single case of acute cauda equina syndrome has been reported [3]. In the present study, the patient had a 2-month history of low back pain exacerbated by a 3-week history of acute partial cauda equina syndrome (sensory disturbance in the perineal and perianal regions, bladder dysfunction). Such a clinical course for chronic SEH has not been previously reported and can thus be considered to be extremely rare.

The histological examination revealed relatively old hemorrhagic changes (hemosiderin deposition), in addition to an early phase change (granulation tissue and blood clot). This implied that a serial bleeding process was responsible for the gradual formation and expansion of the space-occupying lesion and subsequent scalloping. The cauda equina symptoms may have been induced by the recurrence of this bleeding process.

MRI may be considered to be the diagnostic standard for SEHs at a certain level; however, MRI signal intensities of SEHs are variable and depend on the age and stage of biochemical evolution of the hematoma [1, 10, 11]. In the acute phase, SEHs appear isointense when compared with the spinal cord on T1-weighted images; in contrast, they appear hyperintense on T2-weighted images. In the subacute stage, SEHs appear hyperintense on T1-weighted images, whereas on T2-weighted images, they tend to be slightly hyperintense or hypo- to isointense compared with the spinal cord. In the early chronic stage, SEHs are homogeneously hyperintense on both T1- and T2-weighted images. Late chronic SEHs appear isointense compared to the spinal cord on T1-weighted images and very hypointense on T2-weighted images, due to the high levels of ferritin and hemosiderin [1, 2, 11]. A number of previous studies have reported that gadolinium administration slightly enhances the lesion rim, but rarely enhances the chronic SEH itself [2, 3, 11, 12]. In the current case, the chronic SEH had heterogeneous intensity on both T1-weighted and T2-weighted images, while gadolinium T1-weighted images showed a heterogeneous enhancement effect. A heterogeneous signal intensity on plain T1- and T2-weighted images and heterogeneous enhancement after gadolinium injection are considered atypical radiological findings for chronic SEH. To the best of our knowledge, three cases of chronic SEH with a similar MRI signal intensity pattern have been previously described [10, 12]. The atypical MRI signal intensity pattern in the present study was considered to be related to the difficulty of the preoperative diagnosis and history of recurrent bleeding. Moreover, chronic expanding hematomas of the extremities or spine have been reported to have heterogeneous signal intensities on T1- and T2-weighted MRI images, as well as a heterogeneous contrast effect on gadolinium-enhanced MRI. These hematomas gradually increase in size and occasionally erode the adjacent bone. Thus, the differentiation of chronic expanding hematomas from tumoral lesions based on MRI findings is often problematic [13, 14].

It is well known that the increased intraspinal pressure exerted by a slowly expanding intraspinal tumorous lesion may cause scalloping of the spinal osseous structures. Schwannomas, dermoid cysts, epidermoid cysts, and lipomas occasionally demonstrate vertebral scalloping [15–18]. In addition, dumbbell tumors with a dilated intervertebral foramen due to neurogenic tumors (e.g., schwannomas, neurofibromas, and meningiomas) have been widely reported [19, 20]. Benign intraspinal lesions, such as perineural (Tarlov) cysts and herniated discs, are also known to cause vertebral scalloping [21–23]. Vertebral scalloping due to chronic SEHs is extremely rare, with only two cases having been previously reported in the English literature [2, 5]. Although vertebral bone erosion due to SEHs is rare, several cases of bone erosion due to chronic expanding hematomas [13, 24] or hemophilic pseudotumors [25] have been reported to date. Surgeons should be cognizant of the ability of blood-derived space-occupying lesions to cause bone erosion, as well as the use of vertebral scalloping as a diagnostic aid to differentiate chronic SEHs from tumorous lesions.

In conclusion, chronic SEHs are rare clinical entities that can be challenging to diagnosis via preoperative imaging, particularly when associated with vertebral scalloping. Although rare, SEHs should always be considered as a differential diagnosis in patients with suspected tumoral lesions in the spinal canal. Furthermore, surgeons should be aware that chronic SEHs can cause vertebral bone erosion.

## Data Availability

All data generated or analyzed during this study are included in this published article.

## Abbreviations

MRI: Magnetic resonance imaging
SEH: Spinal epidural hematoma
